# Effects of Extended ICU Length of Stay on Braden Scale Validity: A Retrospective Analysis

**DOI:** 10.1097/jnr.0000000000000769

**Published:** 2026-09-10

**Authors:** Yinji JIN, Sun Ho IM, Ji-Sun BACK, Alyoung KIM, JongHyo OH, Sun-Mi LEE

**Affiliations:** 1 School of Nursing, Yanbian University, Jilin, China; 2 College of Nursing, University of Utah, Salt Lake City, UT, USA; 3 Department of Nursing, Dongnam Health University, Suwon, Gyeonggi-do, Republic of Korea; 4 College of Nursing, The Catholic University of Korea, Seoul, Republic of Korea; 5 Department of Neurological Intensive Care Unit, Seoul St. Mary’s Hospital, The Catholic University of Korea, Seoul, Republic of Korea

**Keywords:** Braden scale, intensive care unit, length of stay, pressure injury, validity

## Abstract

**Background:**

Pressure injuries prolong hospitalization, increase costs, and increase mortality risk. Intensive care unit (ICU) patients experience a high prevalence of these injuries due to multiple risk factors. Although the Braden Scale is widely used to predict pressure injury risk, its accuracy in ICU settings is limited, underscoring the need for improvement.

**Purpose:**

This study was designed to evaluate the variance in predictive validity of the Braden Scale for pressure injury development based on ICU length of stay.

**Methods:**

A retrospective analysis was conducted in the medical and surgical ICUs of a university-affiliated hospital in Korea on data from 2,190 patients (419 with and 1,693 without pressure injuries) admitted between January 2017 and February 2020. Predictive validity was assessed using a Braden Scale cutoff of 14 points, and logistic regression was used to analyze the association between risk classification and pressure injury development.

**Results:**

For predicting pressure injuries in patients with ICU stays ≤7 days and ≥8 days, the final Braden Scale score yielded respective Youden Index values of .39 and .18 and respective area under the receiver operating characteristic curve values of .76 and .64. Those with ICU stays ≤7 days and classified as high-risk were more likely to develop pressure injuries than their peers classified as lower risk (odds ratio [*OR*] 7.33, 95% confidence interval [CI; 4.75, 11.31]). Those with ICU stays ≥8 days had an *OR* of 3.04 (95% CI [1.85, 4.98]).

**Conclusions/Implications for Practice:**

The findings of this study support that the accuracy of the Braden scale in terms of predicting pressure injury risk declines as ICU stay length increases. This lower scale performance may be attributable to multiple confounding clinical factors present in ICU settings. To minimize pressure injury risk during extended ICU stays, the Braden Scale should be supplemented with comprehensive assessments of nutritional status, skin perfusion, mobility, and hemodynamic stability, as these indicators more effectively capture patient-specific risks during prolonged hospitalization.

## Introduction

Pressure injuries in hospitalized patients can prolong hospital stays, raise health care expenses, and, in severe instances, increase the risk of mortality (Han et al., 2019; Kim et al., 2024; Ramos-Sánchez et al., 2025). The prevalence of pressure injuries among intensive care unit (ICU) patients ranges from 14% to 62.4% (Bambi et al., 2022; Galetto et al., 2021; VanGilder et al., 2021) and varies by country and demographic factors. This prevalence is significantly higher than the 9%–27% prevalence observed in general wards (Forni et al., 2018; VanGilder et al., 2021).

The elevated prevalence of pressure injuries among ICU patients has been attributed to several interrelated factors. Many ICU patients are transferred from the emergency department or operating room, where prolonged waiting times and extended surgical procedures increase pressure injury risk (Cox et al., 2020). Furthermore, the illness severity of ICU patients is typically higher than that of patients in general wards (Jin et al., 2021). Also, treatment with medications that reduce patient mobility, such as analgesics, antihypertensives, sedatives, and muscle relaxants (Kebapci & Tilki, 2024; Noie et al., 2024; Shui et al., 2021) and inadequate nutritional intake, reliance on ventilators, and use of continuous renal replacement therapy (CRRT; Cox et al., 2020; Pittman et al., 2021; Shui et al., 2021) are associated with higher pressure injury risk. Moreover, the heightened acuity of ICU patients underscores the significance of length of stay (LOS) as a key risk factor (Noie et al., 2024). The results of several studies demonstrate that pressure injury incidence rises markedly over time when ICU stays exceed 2 days up through 14 days (Cox et al., 2020; Galetto et al., 2021).

The accreditation standards established by Joint Commission International recommend using a risk assessment tool with proven validity and reliability to mitigate the incidence of pressure injuries among hospitalized patients. This tool should enable periodic reassessment of all admitted patients at the time of admission, at weekly intervals, and whenever changes in patient condition occur (Joint Commission International, 2008). Several pressure injury risk assessment tools have been developed internationally and in Korea. Although the Braden Scale has been widely adopted for this purpose in Korean medical institutions (Bergstrom et al., 1987), its validity has been shown to vary significantly based on specific hospital characteristics and patient attributes (Huang et al., 2021; Wei et al., 2020). Furthermore, its accuracy when used in ICU settings has been relatively poor, with reported areas under the receiver operating characteristic curve (AUC) ranging from .62 to .80 (Huang et al., 2021; Lee et al., 2021; Wei et al., 2020). The performance of the Braden Scale among adult ICU patients has shown reduced predictive validity, with higher sensitivity but lower specificity (Tao et al., 2024; Wei et al., 2020). These findings highlight the need to either develop new tools or refine existing ones to improve predictive capability (Wei et al., 2020). Moreover, further research is required to identify ICU-specific predictors of pressure injuries, which may help enhance the effectiveness of the Braden Scale among critically ill patients when these risk factors are present.

In previous related studies (Huang et al., 2021; Lee et al., 2021; Wei et al., 2020), the predictive validity of the Braden Scale has been examined using either the initial score at ICU admission or the most recent score immediately before pressure injury occurrence. However, determining the specific post-ICU admission time point that provides the highest predictive value has proven challenging. Furthermore, the predictive values of the Braden Scale have yet to be distinguished in the literature by ICU LOS duration. However, it is important to evaluate predictive value stratified by ICU LOS duration to allow the proactive identification of patients who, although free of pressure injuries at ICU admission, may be at risk of developing these injuries during their stay.

This study was designed to evaluate the predictive validity of the Braden Scale in relation to ICU LOS using electronic medical record (EMR) data. Also, the potential of these findings to guide tailored pressure injury prevention strategies within ICU settings was examined.

The specific objectives of this study were:

1. Identify how pressure injury risk factors, as assessed by the Braden Scale, vary according to ICU LOS duration.
2. Compare the predictive validity of this scale for pressure injury development between ICU patients with short and prolonged stays.
3. Evaluate the performance of different Braden risk classifications in predicting pressure injury by ICU LOS duration.

## Methods

### Study Design

A secondary data analysis approach was employed using information retrospectively extracted from EMR.

### Data Source

**Figure 1:**
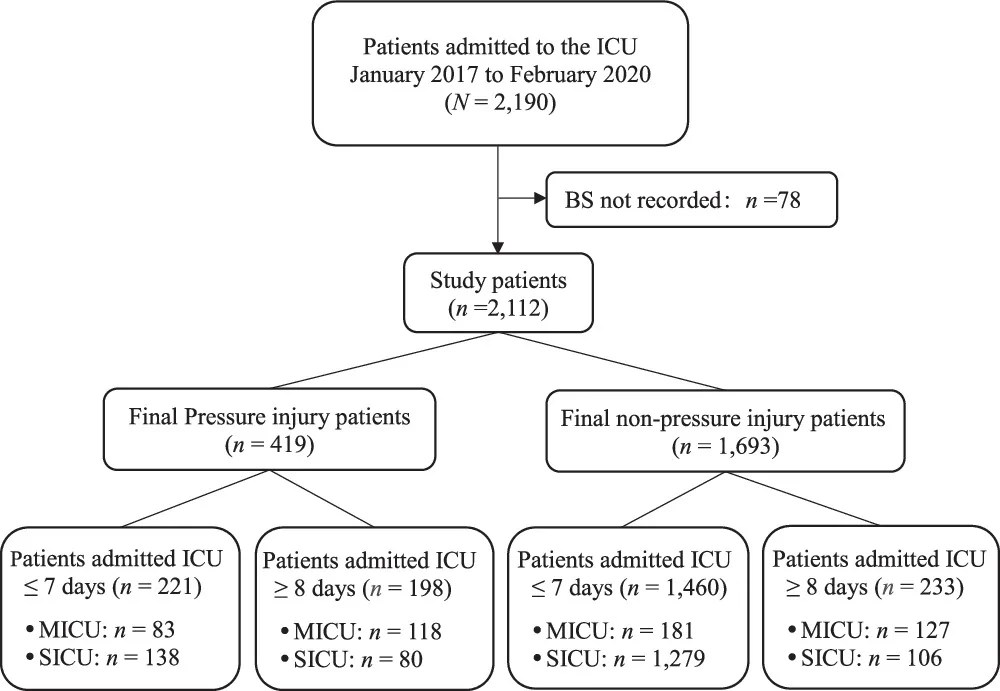
Flow Chart of Study Patient Selection *Note.* ICU = intensive care unit; BS = Braden scale; LOS = length of stay; MICU = medical intensive care unit; SICU = surgical intensive care unit.

The primary study was conducted at a university hospital in Korea with a total of 1,356 beds, of which 40 beds, distributed between the medical intensive care unit (MICU) and surgical intensive care unit (SICU), were selected for this research. The participant pool consisted of 2,190 patients who met the criteria for pressure injury among those admitted to the MICU and SICU between January 2017 and February 2020, as collected for the primary study (Figure 1). Patients classified as having pressure injuries were those who did not exhibit them on the day of admission but were recorded, based on their pressure injury observation records, as having Stage II or higher pressure injuries after the first day of admission. For patients with multiple admissions, only their first admission was considered, resulting in a total of 438 patients with pressure injuries. In addition, nonpressure injury patients admitted to the ICU during the same period as those with pressure injuries were selected. Among these, 1,752 nonpressure injury patients were randomly chosen using a 1:4 ratio (Rigby & Robinson, 2000) from the dataset of patients not included in the pressure injury observation record.

The extracted data included demographic characteristics and risk factors associated with pressure injuries, such as environment/setting, vital signs, nursing severity, laboratory test results, medical treatment, and medication. Nursing severity refers to the patient’s level of nursing need and was assessed daily by the charge nurse, with higher scores indicating greater severity and nursing requirements. Braden Scale scores used in this study were the initial score at admission for patients who developed pressure injuries and their score immediately before pressure injury diagnosis. For patients without pressure injuries, the initial score after admission and the final score before discharge were used.

### Participant Selection

Of the initially considered sample of 2,190 patients, 78 were excluded due to the complete absence of Braden Scale records in their EMR data, resulting in a final sample of 2,112 patients included in the study.

### Measurement Definitions

The Braden Scale, used with permission from the original authors (Bergstrom et al., 1987), comprises six domains: sensory perception, moisture, activity, mobility, nutrition, and friction and shear risk. Among these, friction and shear risk are scored on a scale of 1–3, while the other five domains are scored on a 1–4 scale, with potential total scale scores ranging from 6 (*higher risk of pressure injury*) to 23 (*lower risk*). In this study, patients with a Braden Scale score of 18 or less were classified as at risk of pressure injury.

All of the hospitalized patients in the sample were assessed for pressure injury on admission and once a week thereafter. Reassessments were also performed when the patient’s condition changed (e.g., decreased consciousness, paralysis) and after patient transfer.

If a pressure injury was identified, the responsible nurse verbally reported it to the attending physician, and the head nurse documented the observed injury, consulted wound care specialists, and implemented both targeted interventions and preventive nursing measures.

Preventive care, including repositioning, massage, and skin observation, was implemented at least once per shift. During care, skin cleanliness was maintained with special attention given to incontinent or emaciated bedridden patients. To minimize pressure-related skin damage, appropriate positioning and turning techniques were applied. The head of the bed was not elevated beyond 30° for nonventilated patients. Adequate hydration and a high-protein diet were ensured. Patients and their families were educated on the causes, risks, and prevention of pressure injuries and encouraged to actively participate in care. All preventive measures were documented in the patient’s record.

Pressure injuries were staged using National Pressure Injury Advisory Panel (NPIAP) criteria (National Pressure Ulcer Advisory Panel, 2016), which aligns with the skin assessment tool used in this study, and nurses recorded stage information in a pressure injury registry. To ensure assessment consistency, all of the participating nurses had completed a standardized training program before study implementation. Novice nurses received 2 months of structured ICU training under preceptors, which covered NPIAP staging protocols, case simulations, and standardized patient evaluations. Inter-rater reliability for NPIAP staging, calculated using Cohen’s Kappa, exceeded .90.

Braden Scale measurements were categorized into two distinct time points for analysis. The “first” measurement corresponds to the initial assessment conducted upon admission. The “last” measurement corresponds to either the day before a pressure injury diagnosis (for affected patients) or the last day of hospitalization (for unaffected patients). These two time points were used to compare changes in risk scores during hospitalization and to examine their association with pressure injury development.

### Statistical Analysis

Data analyses were performed using SAS version 9.4 (SAS Institute, Cary, NC, USA). The primary hypothesis tested was that the “last” Braden Scale score indicates a higher pressure injury risk than the “first” score. For continuous variables, missing data (<5% of total observations) were imputed using the mean of the respective variable, as preliminary analyses confirmed missingness to be random and unlikely to bias the results.

Differences in pressure injury risk factors according to LOS were analyzed using *t* tests and χ^2^ tests, with means, standard deviations, and percentages presented for illustration. Logistic regression was used to examine the association between changes in Braden Scale scores and pressure injury occurrence. In the regression analysis, the key demographic/clinical characteristics of age, body mass index (BMI), and activity were incorporated as covariates. Variable selection was based on univariate analysis using a *p* value threshold of <.20 for inclusion in the multivariable model. A multivariable logistic regression model was constructed using a backward stepwise procedure to include clinically relevant variables while maintaining model parsimony. Odds ratios (*OR*s) with 95% confidence intervals (CIs) were reported, and statistical significance was defined as two-sided *p* < .05.

The predictive validity of the Braden Scale was assessed using sensitivity, specificity, positive predictive value (PPV), negative predictive value (NPV), Youden Index (YI), receiver operating characteristic curves, and AUC. The optimal cutoff value of 14 was determined using receiver operating characteristic analysis by maximizing the YI. The cutoff produced an AUC of .78 with a sensitivity of .89 and a specificity of .51 in this study. This aligns with clinical practice guidelines for pressure injury risk stratification that were validated in previous studies with similar patient populations (e.g., ICU patients with prolonged stays), thus balancing accurate risk identification with clinical feasibility.

### Ethical Considerations

This study was approved by the institutional review board (Approval No. KC21RASI1012). All of the participants were assigned a study identification number for data management, and all records were securely stored.

## Results

### Risk Factors of Pressure Injury Patients

Four hundred and nineteen in the study sample were classified as with pressure injury and 1,693 were classified as without pressure injury. The ICU LOS ≤7 days group included 221 patients with pressure injury and 1,460 without, while the ICU LOS ≥8 days group included 198 patients with pressure injury and 233 without (Table 1).

**Table 1:** Demographics and Clinical Characteristics of All Patients (*N*=2,112)

Variable	ICU LOS ≤7 days (*n*=1,681)		χ^2^/*t*	*p*	ICU LOS ≥8 days (*n*=431)		χ^2^ */t*	*p*
	Pressure Injury (*n*=221)	Nonpressure Injury (*n*=1,460)			Pressure Injury (*n*=198)	Nonpressure Injury (*n*=233)		
	*n* (%)	*n* (%)			*n* (%)	*n* (%)		
Age (year; *M* ± *SD*)	67.43±15.65	60.37±16.05	−6.11	<.001	67.28±14.11	65.78±15.18	−1.05	.293
Age ≥65	143 (64.71)	637 (43.63)	34.28	<.001	127 (64.14)	134 (57.51)	1.97	.160
Gender (male)	138 (62.44)	890 (60.96)	−6.11	.673	122 (61.62)	152 (65.24)	0.61	.436
BMI <23 (kg/m^2^)	176 (79.64)	872 (59.73)	32.42	<.001	154 (77.78)	158 (67.81)	5.32	.021
Activity (dependent)	86 (38.91)	164 (11.23)	116.17	<.001	60 (30.30)	53 (22.75)	3.16	.076
Orientation (none)	100 (45.25)	363 (24.86)	39.97	<.001	83 (41.92)	99 (42.49)	0.01	.905
Type of ICU								
MICU	83 (37.56)	181 (12.40)	91.78	<.001	118 (59.60)	127 (54.51)	1.13	.288
SICU	138 (62.44)	1279 (87.60)			80 (40.40)	106 (45.49)		
APACHE II score (*M* ± *SD*)	19.66±8.10	13.80±6.93	−10.22	<.001	20.28±8.14	17.57±6.94	−3.68	<.001
Nursing severity level (Moderate, high) ^a^	179 (81.00)	699 (47.88)	84.38	<.001	195 (98.48)	216 (92.70)	8.08	.005
SBP >140 (mm Hg)	33 (14.93)	316 (21.64)	5.26	.022	23 (11.62)	40 (17.17)	2.64	.104
DBP <60 (mm Hg)	103 (46.61)	448 (30.68)	22.08	<.001	89 (44.95)	110 (47.21)	0.22	.639
Pulse >100 (beat/min)	86 (38.91)	280 (19.18)	43.90	<.001	71 (35.86)	64 (27.47)	3.50	.061
Respiratory rate >25 (breath/min)	32 (14.48)	103 (7.05)	14.33	.001	32 (16.16)	34 (14.59)	0.20	.652
Body temperature >37 (℃)	60 (27.15)	403 (27.60)	0.02	.888	44 (22.22)	67 (28.76)	2.39	.122
Albumin <3 (g/dL)	166 (75.11)	483 (33.08)	143.06	<.001	160 (80.81)	152 (65.24)	12.99	<.001
Hematocrit (%), Male <40; Female <34	200 (90.50)	1070 (73.29)	30.78	<.001	196 (98.99)	221 (94.85)	5.84	.016
CRP >0.47 (mg/L)	187 (84.62)	791 (54.18)	73.09	<.001	188 (94.95)	222 (95.28)	0.03	.874
Total bilirubin >1.58 (mg/dL)	78 (35.29)	284 (19.45)	28.51	<.001	84 (42.42)	76 (32.62)	4.41	.036
BUN >20 (mg/dL)	165 (74.66)	504 (34.52)	129.08	<.001	151 (76.26)	157 (67.38)	4.14	.042
Infection	74 (33.48)	170 (11.64)	73.79	<.001	72 (36.36)	61 (26.18)	5.20	.023
CRRT	43 (19.46)	76 (5.21)	59.27	<.001	63 (31.82)	35 (15.02)	17.19	<.001
Ventilator	120 (54.30)	176 (12.05)	236.10	<.001	158 (79.80)	140 (60.09)	19.49	<.001
Foley cathetherization	199 (90.05)	1329 (91.03)	0.22	.636	174 (87.88)	221 (94.85)	6.80	.009
Restraints	145 (65.61)	291 (19.93)	208.50	<.001	182 (91.92)	170 (72.96)	25.70	<.001

*Note.* ICU = intensive care unit; LOS = length of stay; BMI = body mass index; MICU = medical intensive care unit; SICU = surgical intensive care unit; APACHE II = acute physiology and chronic health evaluation II; SBP = systolic blood pressure; DBP =diastolic blood pressure; CRP = C-reactive protein; BUN = blood urea nitrogen; CRRT = continuous renal replacement therapy.

^a^ Nursing severity level = nursing need score, moderate = 92~141, high =142~999.

Pressure injury patients accounted for 13.15% of those hospitalized for ≤7 days and 45.94% of those hospitalized for ≥8 days. The mean age of pressure injury patients in the ≤7 days group was 67.43±15.65 years, which is significantly higher than that of nonpressure injury patients (60.37±16.05 y; *p* < .05). Nursing severity during the first 7 days of admission was 81% for pressure injury patients and significantly less (47.88%) for nonpressure injury patients (*p* < .05). For those hospitalized for ≥8 days, the corresponding figures were 98.48% and 92.70%, respectively, again showing statistical significance (*p* <.05).

### Predictive Validity of the Braden Scale

The 2,112 patients in the study sample were assessed 3,548 times using the Braden Scale, with all assessed between 1 and 16 times for an average of 1.64 times per patient. Patients with pressure injuries were assessed an average of 1.14 times, while those without pressure injuries were assessed an average of 2.32 times. Regarding the predictive validity of the Braden Scale after hospitalization, for all patients, the tool demonstrated a sensitivity of .75, specificity of .57, PPV of .30, and NPV of .90. For the final Braden Scale assessment, the sensitivity was .89, specificity was .51, PPV was .31, and NPV was .95 (Table 2).

**Table 2:** Braden Scale Predictive Validity (*N*=2,112)

Classification	Time	TP	FN	FP	TN	Sensitivity	Specificity	PPV	NPV	YI	Accuracy	AUC
All patients (*N*=2,112)	First	314	105	732	961	.75	.57	.30	.90	.32	.60	.70
	Last	372	47	836	857	.89	.51	.31	.95	.39	.58	.78
Patients ≤7 d (*n*=1,681)	First	169	52	592	868	.76	.59	.22	.94	.36	.62	.72
	Last	186	35	660	800	.84	.55	.22	.96	.39	.59	.76
Patients ≥8 d (*n*=431)	First	145	53	140	93	.73	.40	.51	.64	.13	.55	.60
	Last	186	12	176	57	.94	.24	.51	.83	.18	.56	.64
MICU (*n*=509)	First	169	32	212	96	.84	.31	.44	.75	.15	.52	.64
	Last	191	10	248	60	.95	.19	.44	.86	.15	.49	.65
SICU (*n*=1,603)	First	145	73	520	865	.67	.62	.22	.92	.29	.63	.67
	Last	181	37	588	797	.83	.58	.24	.96	.41	.61	.76
MICU LOS ≤7 d (*n*=264)	First	73	10	127	54	.88	.30	.37	.84	.18	.48	.68
	Last	77	6	141	40	.93	.22	.35	.87	.15	.44	.68
SICU LOS ≤7 d (*n*=1,417)	First	96	42	465	814	.70	.64	.17	.95	.33	.64	.69
	Last	109	29	519	760	.79	.59	.17	.96	.38	.61	.74
MICU LOS ≥8 d (*n*=245)	First	96	22	85	42	.81	.33	.53	.66	.14	.56	.62
	Last	114	4	107	20	.97	.16	.52	.83	.12	.55	.59
SICU LOS ≥8 d (*n*=186)	First	49	31	55	51	.61	.48	.47	.62	.09	.54	.55
	Last	72	8	69	37	.90	.35	.51	.82	.25	.59	.69

*Note.* TP = true positive; FN = false negative; FP = false positive; TN = true negative; PPV = positive predictive value; NPV = negative predictive value; YI = Youden index; AUC = area under the receiver operating characteristic curve; d = day; MICU = medical intensive care unit; SICU = surgical intensive care unit. First = the initial Braden scale score after admission; Last = last Braden scale score before pressure injury occur or discharge.

When stratified by LOS, within 7 days of admission, the YI and AUC for all of the patients were .39 and .76 at the final assessment, respectively, exceeding the .36 and .72 observed at the initial assessment.

For the subset of patients hospitalized for ≥8 days, the YI and AUC at the final assessment were .18 and .64, respectively, compared with .13 and .60 at the initial assessment.

Predictive performance was stronger in SICU patients than MICU patients, with final assessment YI and AUC values significantly higher for SICU patients than MICU patients (.41/.76 vs. .15/.65, respectively). Although predictive accuracy declined in both ICU settings for patients with stays ≥8 days, the decrease was more pronounced among those in the MICU, with SICU patients showing greater improvement across all subgroups, particularly among those with ≥8 days of hospitalization, where metrics (.25/.69) substantially exceeded those of MICU patients (.12/.59).

### Logistic Regression Analysis of the Braden Scale Risk Classification by LOS Category

In this study, patients initially categorized as normal or low-risk were grouped into the “low-risk” category, moderate-risk patients remained in the “moderate-risk” category, and those categorized as high or very high risk were combined into the “high-risk” category (Table 3). During the univariate analysis, the distribution of pressure injury patients across these categories at baseline revealed that 24.58% were classified as high risk, 31.27% as moderate risk, and 44.15% as low risk. In contrast, among nonpressure injury patients, 5.55% were classified as high risk, 18.84% as moderate risk, and 75.61% as low risk. The between-group differences were statistically significant (*p* < .05). The logistic regression analysis of data from the full sample showed significant outcomes, with high-risk patients associated with an *OR* of 9.88 (95% CI: [7.15, 13.64]) and moderate-risk patients with an *OR* of 3.57 (95% CI [2.72, 4.70]) at the final time point, both of which reached statistical significance (*p* < .001).

**Table 3:** Logistic Regression Analysis of Braden Scale Risk Classification for Pressure Injury, by Length of Stay

Time	BS Risk Classification	All Patients (*N*=2,112)			ICU LOS ≤7 d (*n*=1,681)			ICU LOS ≥8 d (*n*=431)		
		Pressure Injury (*n*=419)	Nonpressure Injury (*n*=1,693)	*OR* (95% CI)	Pressure Injury (*n*=221)	Nonpressure Injury (*n*=1,460)	*OR* (95% CI)	Pressure Injury (*n*=198)	Nonpressure Injury (*n*=233)	*OR* (95% CI)
First	High risk	103 (24.58)	94 (5.55)	5.38 [3.84, 7.54]***	53 (23.98)	64 (4.38)	6.38 [4.08, 9.97]***	50 (25.25)	30 (12.88)	2.49 [1.44, 4.29]***
	Moderate risk	131 (31.27)	319 (18.84)	2.28 [1.74, 2.97]***	70 (31.68)	256 (17.54)	2.33 [1.64, 3.33]***	61 (30.81)	63 (27.03)	1.50 [.96, 2.35]
	Low risk	185 (44.15)	1,280 (75.61)	1	98 (44.34)	1,140 (78.08)	1	87 (43.94)	140 (60.09)	1
	C statistics			.74			.77			.62
Last	High risk	147 (35.08)	139 (8.21)	9.88 [7.15, 13.64]***	63 (28.51)	74 (5.07)	7.33 [4.75, 11.31]***	84 (42.42)	65 (27.90)	6.26 [3.65, 10.74]***
	Moderate risk	153 (36.52)	364 (21.50)	3.57 [2.72, 4.70]***	76 (34.39)	282 (19.31)	2.72 [1.91, 3.88]***	77 (38.89)	82 (35.19)	3.04 [1.85, 4.98]***
	Low risk	119 (28.40)	1,190 (70.29)	1	82 (37.10)	1,104 (75.62)	1	37 (18.69)	86 (36.91)	1
	C statistics			.79			.78			.71

*Note.* BS = Braden scale; ICU = intensive care unit; LOS = length of stay; *OR* = odds ratio; CI = confidence interval; C = concordance statistics. First = the initial Braden scale score after ICU admission; Last = last Braden scale score before pressure injury occur or discharge; Braden scale score of 18 or less was considered a risk group for pressure injury, and the highest-risk group was classified as 6–9, the high-risk group as 10–12, the moderate risk group as 13–14, the low-risk group as 15–18, and the normal group as 19–23; high-risk = highest-risk and high-risk group; moderate-risk = moderate-risk group; low-risk = low-risk and normal group. Covariates: age, BMI, activity; the univariate of all variables was statistically significant (*p*<.05).

***p < .001.

### Characteristics of Patients’ Combined Final Data Before Pressure Injury

A high NPV = .90–.96 indicates the Braden Scale effectively rules out risk, while a low PPV = .22–.31 reflects a higher false-positive rate. When comparing the difference between agreement and disagreement based on the assessment conducted immediately prior to a pressure injury diagnosis, of the 1,681 patients with LOS ≤7 days, 986 showed agreement and 695 disagreement between predicted and observed outcomes; of the 431 patients with LOS ≥8 days, 243 were in agreement and 188 in disagreement (Table 4). For those with LOS ≥8 days, patients in disagreement were older than those in agreement and had higher body temperatures (>37 °C). Foley catheter use was significantly less frequent among patients in disagreement (*p* < .05).

**Table 4:** Patient Data Characteristics, Assessment Immediately Before Pressure Injury Diagnosis (*N*=2,112)

Variable	Length of Stay ≤7 days (*n*=1,681)		χ^2^ */t*	*p*	Length of Stay ≥8 days (*n*=431)		χ^2^ */t*	*p*
	Agreement (*n*=986)	Disagreement (*n*=695)			Agreement (*n*=243)	Disagreement (*n*=188)		
	*n* (%)	*n* (%)			*n* (%)	*n* (%)		
Age (years; *M*±*SD*)	60.66±16.12	62.19±16.22	1.91	.056	65.23±14.64	68.06±14.66	1.99	.047
Age ≥65	445 (45.13)	335 (48.20)	1.54	.214	140 (57.61)	121 (64.36)	2.02	.155
Gender (male)	602 (61.05)	426 (61.29)	0.01	.921	157 (64.61)	117 (62.23)	0.26	.611
BMI <23 (kg/m^2^)	606 (61.46)	442 (63.60)	0.79	.373	178 (73.25)	134 (71.28)	0.21	.649
Activity (dependent)	126 (12.78)	124 (17.84)	8.25	.004	58 (23.87)	55 (29.26)	1.59	.207
Orientation (none)	232 (23.53)	231 (33.24)	19.25	<.001	102 (41.98)	80 (42.55)	0.01	.904
APACHE II score (*M*±*SD*)	13.81±6.82	15.64±7.94	4.93	<.001	19.09±87.98	18.46±7.15	−0.84	.399
Nursing severity level (moderate, high) ^a^	501 (50.81)	377 (54.24)	1.93	.165	229 (94.24)	182 (96.81)	1.58	.209
SBP >140 (mm Hg)	199 (20.18)	150 (21.58)	0.49	.486	29 (11.93)	34 (18.09)	3.21	.073
DBP <60 (mm Hg)	327 (33.16)	224 (32.23)	0.16	.688	117 (48.15)	82 (43.62)	0.88	.349
Pulse >100 (beat/min)	173 (17.55)	193 (27.77)	25.02	<.001	78 (32.1)	57 (30.32)	0.16	.693
Respiratory rate >25 (breath/min)	65 (6.59)	70 (10.07)	6.68	<.001	34 (13.99)	32 (17.02)	0.75	.386
Body temperature >37 (℃)	240 (24.34)	223 (32.09)	12.25	<.001	49 (20.16)	62 (32.98)	9.10	.003
Albumin <3 (g/dL)	352 (35.70)	297 (42.73)	8.51	.004	172 (70.78)	140 (74.47)	0.72	.396
Hematocrit (%) male <40, female <34	745 (75.56)	525 (75.54)	0.00	.993	237 (97.53)	180 (95.74)	1.08	.300
CRP >0.47 (mg/L)	517 (52.43)	461 (66.33)	32.36	<.001	229 (94.24)	181 (96.28)	0.95	.330
Total bilirubin >1.58 (mg/dL)	186 (18.86)	176 (25.32)	10.07	<.001	97 (39.92)	63 (33.51)	1.86	.172
BUN >20 (mg/dL)	378 (38.34)	291 (41.87)	2.12	.145	178 (73.25)	130 (69.15)	0.87	.350
Infection	154 (15.62)	90 (12.95)	2.34	.126	78 (32.1)	55 (29.26)	0.40	.526
CRRT	47 (4.77)	72 (10.36)	19.39	<.001	63 (25.93)	35 (18.62)	3.22	.073
Ventilator	126 (12.78)	170 (24.46)	38.34	<.001	171 (70.37)	127 (67.55)	0.39	.530
Foley cathetherization	898 (91.08)	630 (90.65)	0.09	.764	216 (88.89)	179 (95.21)	5.54	.019
Restraints	196 (19.88)	240 (34.53)	45.57	<.001	198 (81.48)	154 (81.91)	0.01	.908

*Note.* BMI = body mass index; APACHE II = acute physiology and chronic health evaluation II; SBP = systolic blood pressure; DBP = diastolic blood pressure; CRP = C-reactive protein; BUN = blood urea nitrogen; CRRT = continuous renal replacement therapy.

^a^ Nursing severity level = nursing need score, moderate = 92~141; high = 142~999.

## Discussion

In this study, EMRs were used to record the results of the pressure injury risk assessments performed by nurses using the Braden Scale. To evaluate scale validity, patients’ LOS were stratified by MICU and SICU status. The analysis in this study considered Braden scores throughout hospitalization, including the first score after admission and the last score before pressure injury occurrence, using a cutoff value of 14 points.

As hypothesized, the predictive validity of the final assessment exceeded that of the initial assessment. For the full sample, average sensitivity and NPV were .89 and .95, respectively, at the last assessment. These figures were higher than those reported in earlier studies, where sensitivity values ranged from .50 to .78 (Huang et al., 2021; Theeranut et al., 2021; Yuan et al., 2023) and the NPV was .93 (Theeranut et al., 2021), indicating robust performance in this study for both parameters.

For the full sample, the AUC reached .78 during the final assessment, exceeding the range reported in prior investigations (.65–.77; Delawder et al., 2021; Theeranut et al., 2021). When predictive validity was examined separately for MICU and SICU patients, the predictive capacity for SICU patients was superior to that for MICU patients. Specifically, the AUC at the final measurement before pressure injury onset in the SICU was .76, which was higher than the .71 reported in a previous study of predictive tools (Tao et al., 2024) and consistent with the findings from a Turkish SICU cohort (Gurkan et al., 2022).

Across the entire cohort and within both MICU and SICU subgroups, the AUC declined more for patients hospitalized for ≥8 days compared with those with stays ≤7 days. This pattern, which shows a reduction in Braden Scale predictive accuracy with ICU LOS, indicates that extended hospitalization may diminish the ability of this scale to anticipate pressure injury risk.

In comparing pressure injury and nonpressure injury patients by LOS (≤7 d vs. ≥8 d), several clinical and hematological indicators were shown to be significantly associated with pressure injury risk, which is consistent with previous studies (Cox et al., 2020; Lee et al., 2021; Pittman et al., 2021). Severity indices included the Acute Physiology and Chronic Health Evaluation (APACHE) II (0–71 points; higher = more severe) and nursing severity level (moderate: 92–141, high: 142–999; higher = greater care needs). Hematological indicators referred to albumin (normal ≥3 g/dL) and hematocrit (normal ≥40% for males and ≥34% for females).

As shown in Table 1, the LOS ≤7 days group had a higher average APACHE II score (19.66±8.10 vs. 13.80±6.93, *p* <.001), higher proportion of moderate or high nursing severity (81.00% vs. 47.88%, *p* < .001), lower average albumin level (<3 g/dL: 75.11% vs. 33.08%, *p* < .001), and lower average hematocrit level (90.50% vs. 73.29%, *p* < .001) than those patients without pressure injuries. In the LOS ≥8 days group, pressure injury patients continued to show higher APACHE II scores (20.28±8.14 vs. 17.57±6.94, *p* < .001) and lower albumin levels (<3 g/dL: 80.81% vs. 65.24%, *p* < .001). These narrower group differences confirm that prolonged LOS correlates with increased illness severity and poorer physiological status, which are key contributors to pressure injury risk. Notably, modifiable factors such as having low albumin and hematocrit levels and using CRRT, ventilators, and restraints were more common in patients with longer LOS durations in both groups, but were significantly more prevalent in patients with pressure injuries (Table 1). In the LOS ≤7 days group, 19.46% of pressure injury patients received CRRT compared with 5.21% of those without injuries (*p* < .001). Ventilation use was 54.30% in injury patients versus 12.05% in noninjury patients (*p* < .001). In the LOS ≥8 days group, 31.82% of pressure injury patients received CRRT versus 15.02% (*p* < .001), and 79.80% used ventilators versus 60.09% (*p* < .001). Because of the retrospective observational nature of this study, causality cannot be inferred; rather, these findings further reveal that mechanical ventilation, CRRT, and physical restraints are associated with higher pressure injury risk, primarily because sedation and restraints limit patient mobility (Celik et al., 2023; Cox et al., 2020). Also, the logistic regression results (Table 3) and predictive validity data (Table 2) indicate predictive performance of the scale declined with LOS duration. In the LOS ≤7 days group, high-risk patients identified using the Braden Scale had an *OR* of 7.33 (95% CI [4.75, 11.31], *p* < .001), while those in the LOS ≥8 days group had a significantly lower OR of 3.04 (95% CI [1.85, 4.98], *p* < .001). Correspondingly, the YI decreased from .39 to .18, and the AUC declined from .76 to .64.

Therefore, systematic risk assessment combining the Braden Scale with continuous monitoring of albumin and hematocrit levels and documentation of medical equipment use is essential for ICU patients with extended LOS durations. This integrated approach compensates for the reduced predictive accuracy of the Braden Scale and addresses the higher prevalence of modifiable risk factors in this population.

Upon analyzing pressure injury risk according to Braden Scale classifications, both high- and moderate-risk categories showed greater susceptibility to pressure injuries. This observation, which aligns with prior research, demonstrates that lower Braden Scale scores correspond to higher pressure injury risk (Cox et al., 2020; Galetto et al., 2021). In the high-risk category, the initial assessment results revealed that pressure injury risk declined sequentially across all patient groups (i.e., those with LOS ≤7 days, all patients combined, and those with LOS ≥8 days). However, in the final assessment, risk revealed a declining trend as LOS increased. Specifically, within the high-risk group, patients with stays ≥8 days exhibited a lower observed risk than those with stays ≤7 days. This pattern supports the finding that predictive validity declined as LOS duration increased, suggesting a gradual reduction in Braden Scale predictive efficacy over time.

When comparing disagreement (false positive/false negative) and agreement (true positive/true negative) in Braden Scale predictions at the last assessment among ICU patients with prolonged stays (LOS ≥8 d, *n*=431), notable objective differences emerged between the groups. The disagreement group (*n*=188) was significantly older (68.06±14.66 vs. 65.23±14.64 y, *p* = .047), had higher average body temperature readings >37 > (32.98% vs. 20.16%, *p* = .003), and exhibited higher Foley catheter use (95.21% vs. 88.89%, *p* = .019) than the agreement group (*n*=243).

These discrepancies highlight two main limitations of the Braden Scale when applied to patients with prolonged ICU stays. Predictive disagreement indicates two specific weaknesses: (a) subjectivity in scoring: Clinical judgments of items such as mobility are often inconsistent, particularly in units like the MICU, where patients are frequently bedridden. This misalignment with objective indicators such as catheter use can reduce predictive accuracy (Cox et al., 2020). (b) Infrequent reassessment: Routine or triggered Braden assessments may fail to capture acute clinical changes such as fever or the introduction of new medical devices, which are common among patients with extended stays (Cobos-Vargas et al., 2024; Jin et al., 2021). For example, elevated temperature, a known risk factor for pressure injury, was more common in the disagreement group but not reflected in Braden scores, contributing to false predictions.

Our findings align with a number of studies (e.g., Ingleman et al., 2024; Mehicic et al., 2024; Wei et al., 2020) that also reported reduced Braden Scale validity in complex, prolonged ICU cases and emphasized the importance of incorporating continuous, objective monitoring to complement the scale in this high-risk population.

Furthermore, patients with lower BMI values and albumin levels below 3 g/dL, both of which are indicators of nutritional status, were more prevalent among patients with pressure injuries relative to those without pressure injuries. This finding underscores the role of nutritional status in pressure injury risk and aligns with prior research identifying nutrition as a major pressure injury risk factor (Kim et al., 2024; Lee et al., 2021; Zhang et al., 2024). The results suggest that extended periods of weight loss and hypoalbuminemia may increase susceptibility to pressure injuries. Therefore, alongside the Braden Scale, ICU nurses should continuously monitor patient weight, vital signs, and hematological parameters (particularly albumin and hematocrit) to gain objective insight into the risk factors of pressure injuries. Ongoing surveillance remains essential, as pressure injuries may occur even in patients categorized as low risk. Continuous reassessment and targeted interventions should thus be implemented to ensure comprehensive prevention.

### Limitations

This study was conducted retrospectively using the EMR data of 2,112 patients from the ICU of a single tertiary general hospital in Korea. Thus, caution is warranted when generalizing the findings. The single-institution design and limited sample scope may not capture rare pressure injury risk profiles, thereby restricting applicability beyond Korean ICUs and nontertiary care settings.

In addition, as this was a secondary data analysis of retrospectively collected EMR data, researchers were not able to predefine or control for all desired variables. For example, potential factors of influence on Braden Scale measurements, such as nurse age and clinical experience, were unavailable and thus may have introduced unmeasured bias.

Furthermore, the high variability in ICU patient characteristics over time highlights the need to develop a pressure injury assessment tool that integrates variables relevant to evolving risk profiles and is adaptable to each patient’s condition and department.

### Conclusions

This study provides evidence regarding how ICU LOS influences the predictive validity of the Braden Scale that may be used in optimizing pressure injury risk assessments in Korean MICUs and SICUs. Notably, the validity of this scale was found to decline with hospitalization duration, showing higher sensitivity (.89) but lower specificity (.51) in ICUs.

To strengthen pressure injury prevention amid frequent patient condition changes, the Braden Scale should be used alongside objective indicators such as age, body temperature, catheter use, and albumin levels and paired with targeted interventions. For patients with LOS ≥8 days, repositioning should occur every 2 hr, pressure-redistributing mattresses should be used, MICU staff should align subscale scoring with immobility indicators, and early nutritional support should be initiated specifically for patients with albumin <3 g/dL.
